# Patient-Reported Outcomes for Quality of Life in SLE: Essential in Clinical Trials and Ready for Routine Care

**DOI:** 10.3390/jcm10163754

**Published:** 2021-08-23

**Authors:** Matthew H. Nguyen, Frank F. Huang, Sean G. O’Neill

**Affiliations:** 1 Liverpool Hospital, Liverpool, NSW 2170, Australia; Matthew.nguyen4@gmail.com; 2 Pathology Department, School of Medical Sciences, Faculty of Medicine, University of New South Wales, Kensington, NSW 2052, Australia; 3 Rheumatology Department, Royal North Shore Hospital, St Leonards, NSW 2065, Australia; fhuangfrank@gmail.com; 4 Northern Clinical School, Faculty of Medicine and Health, University of Sydney, St Leonards, NSW 2065, Australia

**Keywords:** systemic lupus erythematosus, health-related quality of life, patient-reported outcomes, clinical follow-up, outcome measures

## Abstract

Patient-reported outcome (PRO) instruments are widely used to assess quality of life in Systemic Lupus Erythematosus (SLE) research, and there is growing evidence for their use in clinical care. In this review, we evaluate the current evidence for their use in assessing quality of life in SLE in both research and clinical settings and examine the different characteristics of the commonly used PRO tools. There are now several well-validated generic and SLE-specific tools that have demonstrated utility in clinical trials and several tools that complement activity and damage measures in the clinical setting. PRO tools may help overcome physician–patient discordance in SLE and are valuable in the assessment of fibromyalgia and type 2 symptoms such as widespread pain and fatigue. Future work will identify optimal PRO tools for different settings but, despite current limitations, they are ready to be incorporated into patient care.

## 1. Introduction

Systemic Lupus Erythematosus (SLE) is a chronic autoimmune condition that can lead to inflammatory damage of multiple organ systems with clinical manifestations varying from patient to patient [1]. Many patients experience a significantly reduced health-related quality of life (HRQoL), citing fatigue, widespread pain and depression among the most common and debilitating features of the disease [2,3]. It is now well recognised that focusing on disease activity and damage does not allow a physician to adequately quantify or address the patient experience of the disease [3,4]. Good disease control does not guarantee improved quality of life in SLE patients, and failure to address this concern may contribute to treatment non-adherence and/or interruptions [5,6]. Numerous groups now advocate for the measurement of HRQoL and the use of patient-reported outcome (PRO) measures in SLE clinical trials and increasingly in routine care, including the American College of Rheumatology (ACR) [7], the European Alliance of Associations for Rheumatology (EULAR) [8], the Outcome Measures in Rheumatology Clinical Trials (OMERACT) [9] and the World Health Organisation (WHO) [10].

Despite the discordance between physician and patient assessment of SLE, literature on HRQoL is still sparse compared to that on disease activity, organ damage and immunotherapeutics in SLE [11]. A major reason behind the suboptimal focus on HRQoL in SLE management is the fact that HRQoL is a multi-dimensional concept without a concrete definition [12]. If clinicians were to enquire about the factors that patients perceive to be most relevant to their HRQoL, these would be significantly variable. However, overarching themes pertaining to HRQoL have been established, including the perceived impacts of disease and its treatment on physical, emotional and social functioning [10,12]. A variety of PRO tools have been used around the world in both clinical trials and routine care to help clinicians assess and monitor HRQoL in SLE patients [13,14]. Lack of agreement on which PRO to utilise along with real and perceived difficulties in their use in the clinical setting has seen a poor uptake of these tools, even though their routine use was recommended by OMERACT more than 20 years ago [9,15,16].

This focused review will consider the role of various generic and SLE-specific HRQoL tools and their utility in both clinical trials and routine care. To date, SLE-specific tools include Lupus Quality of Life (LupusQoL), Lupus Patient-Reported Outcome (LupusPRO), SLE-Specific Quality of Life (SLEQoL), Lupus Quality of Life (L-QoL) and Lupus Impact Tracker (LIT). All these tools have been validated for use in SLE patients but differ in terms of their item numbers and the domains they encompass [13,14]. Conversely, generic PRO tools were not designed to measure HRQoL in any specific disease population. However, some of these tools including the 36-item Short-Form Health Survey (SF-36) and the EuroQoL-5D (EQ-5D) have been widely utilised in SLE research due to their domains aligning with those relevant to Lupus-related QoL. Generic PRO tools also have the advantage of enabling comparison with other disease states. Currently, there are no clear guidelines or evidence to help clinicians determine what is an optimal PRO, as well as in which specific contexts [10,13].

Recently, two generic HRQoL instruments, Multi-Dimensional Health Assessment Questionnaire (MDHAQ) and Patient-Reported Outcomes Measurement Information System (PROMIS) are gaining traction, with increasing studies demonstrating their validity and utility in measuring HRQoL within the SLE population [13]. As they are not disease-specific, these tools have greater potential in allowing for comparisons with other disease populations or sub-cohorts within the SLE population such as patients with concomitant fibromyalgia or newly termed features of “type 2 SLE”. This is clinically useful, as patients with these symptoms are less responsive to traditional immunosuppressive agents [17]. In addition, these tools have multiple benefits for clinicians beyond measuring HRQoL. For example, MDHAQ can be used to screen for Fibromyalgia (FAST3/FAST4 score) [18] and assess patient’s flare status (RAPID3 score) [19,20]. Specific PRO tools have also been used to assess SLE disease activity and damage [8,15]. In this review, we will provide a summary of the major PRO tools that have been explored for the assessment of HRQoL in SLE research and clinical practice.

## 2. SLE-Specific PRO Tools

### 2.1. LupusQoL

LupusQoL is an SLE-specific instrument measuring HRQoL that has undergone extensive validation in the UK and has been widely adapted to other cohorts [21]. It has been validated in a US sample of SLE patients [22] and also cross-culturally with cohorts from Spain [23], Iran [24], Turkey [25], Italy [26], France [27], Venezuela [28] and China [29]. LupusQoL is a 34-item questionnaire that covers eight domains including physical health, pain, planning, intimate relationships, burden to others, emotional health, body image and fatigue. The recall period for each item is the preceding four weeks, and responses are given on a 5-point Likert scale. The summary score is reported on a scale from 0 to 100, with higher values indicating overall better HRQoL [21,30].

Draft items of this instrument were generated through the identification of recurring themes in qualitative interviews with 30 SLE patients, alongside input from clinical experts [30]. These were then re-assessed by 20 SLE patients whose feedback was incorporated to form the current questionnaire. Although information on readability was not provided, the tool was shown to have good internal consistency, test–retest reliability, concurrent validity and responsiveness to changes with patient-reported deterioration or improvement in health status [31]. However, only six of eight LupusQoL domains were found to be sensitive to improvement of disease activity, and none to deterioration. Floor effects (an inability of the PRO tool to detect true differences in HRQoL at the low end of the scale and below) and ceiling effects (an inability of the instrument to identify true differences in HRQoL at the high end of the scale and above) were mostly acceptable, aside from the intimate relationships and planning domains [30].

Two studies (McElhone [32] and Devilliers [33]) have established definitions for minimal clinically important difference (MCID) for LupusQoL domains. Using anchor-based analysis, McElhone’s study determined that domain MCIDs ranged from −2.4 to −8.7 for deteriorations and from 3.5 to 7.3 for improvements [32]. Devilliers’ study used a similar approach and reported MCIDs ranging from −0.5 to −6.4 for deteriorations and from 1.1 to 9.2 for improvements [33]. Nantes and colleagues showed in a prospective study comprised of 78 disease-active SLE patients that the percentages of patients reporting changes (improvements or deteriorations) across domains varied between MCID definitions, with percentages for most domains being greater using Devilliers’ definition [34].

To date, LupusQoL has been used in five randomised–controlled clinical trials (RCTs). Two Phase III trials assessing the efficacy and safety of Epratuzumab in SLE patients with moderate-to-severe disease found no significant differences in various disease activity scores or LupusQoL scores between the placebo and the treatment groups, at 48 weeks [35]. Moreover, a Phase 4 multi-centre RCT examining the efficacy and safety of Acthar Gel in persistently active SLE patients demonstrated significant and clinically meaningful improvements in LupusQoL scores for the pain, planning, and fatigue domains in those who had higher disease activity levels [36]. Lastly, the remaining two clinical trials found that upper limb exercises [37] and a digital therapeutic plus telehealth coaching intervention [38] led to significant and clinically meaningful improvements in HRQoL as measured by LupusQoL scores. LupusQoL was not designed for use in the clinical setting and has not been studied as a PRO for use in routine care.

### 2.2. LupusPRO

LupusPRO is another SLE-specific tool that was developed in the United States to account for the ethnically diverse population of SLE patients within this demographic [39]. It also incorporated feedback from both genders in its development and is written in gender-neutral language. The tool itself is a 43-item questionnaire that encompasses not only HRQoL domains such as lupus symptoms, cognition and body image but also non-HRQoL domains including desires–goals, coping, social support and satisfaction with care. For each item, patients respond using a 5-point Likert scale, and the total score will range from 0 (worst QoL) to 100 (best QoL). Developers of LupusPRO have proposed that, beyond its ability to assess QoL longitudinally, it is a useful screening tool to help clinicians in determining important aspects of QoL (both health- and non-health related) which could be addressed through initiating discussion or making appropriate referrals. Another advantage is that it was created using recurring themes through patient feedback, and thus the questionnaire is fairly SLE-specific and simple to comprehend [39]. However, the feasibility of using this tool has not been formally evaluated, but this questionnaire would likely be less favourable for use in busy clinics due to its relatively higher number of items [15].

In an inception cohort comprising 323 SLE patients, adequate internal consistency and reliability were found in all domains except for the lupus medication domain [39]. Test–retest reliability was overall fair but particularly lower in some non-HRQoL domains and the procreation domain. Construct validity of the LupusPRO was established through its strong correlations with domains of SF-36, and criterion validity was demonstrated through its correlations with various disease activity and damage measures [39]. It has been validated in several different languages including Tagalog (Philippines) [40], Turkish (Turkey) [41], Spanish (Spain) [42], French (Canada) [43], Italian (Italy) [44], Japanese (Japan) [45], Hindi (India) [46], Arabic (Egypt) [47] and Chinese (Hong Kong) [48]. LupusPRO was found to be valid and reliable within these populations and showed measurement equivalence. Interestingly, LupusPRO was shown to perform similarly across two differing samples of SLE patients, one of which included an ethnically diverse urban cohort from Southern California, whilst the other comprised an ethnically homogeneous rural cohort from the Philippines using confirmatory factor analysis [49]. This demonstrates measurement equivalence for LupusPRO across ethnically diverse and homogeneous populations.

Another advantage of LupusPRO is that it included patients with concomitant fibromyalgia in its design to improve generalisability for the fatigue domain [39]. Recently, an updated version of LupusPRO (LupusPRO v1.8) [50] was developed, in which the Pain–Vitality domain was separated into three domains including sleep, pain and vitality and was captured through the addition of six further items. This updated version demonstrates acceptable face, content, convergent, discriminant and criterion validity with acceptable internal consistency and reliability in all domains, except procreation and coping. LupusPRO has only been used in two clinical trials to date [51,52]. One examined the efficacy of an online training program focused on development of pain-coping skills (PainTRAINER) in SLE Patients and reported meaningful improvements in LupusPRO HRQoL scores in patients who received the intervention compared to those in the wait-list control group at 9 weeks [51]. The other trial demonstrated significant improvements in LupusPRO body image (BI) scores in patients with cutaneous involvement who received a novel BI intervention that used a cognitive-based therapy approach compared to those who did not. There was also significant improvement in scores of other HRQoL domains including pain–vitality, cognition and lupus symptoms post-intervention compared to baseline within the intervention group [52].

### 2.3. SLEQoL

SLEQoL is a 40-item questionnaire that was developed and validated in an English-speaking cohort of SLE patients in Singapore [21,53]. Items were originally generated by rheumatology experts and then modified according to feedback from 100 patients for content validity. Responses for each item are given on a 7-point response scale and capture the patient’s experience over the preceding week. Items encompass six domains including physical functioning, activities, symptoms, treatment, mood and self-image. The summary score is derived from the sum of all responses across these domains and ranges from 40 to 280, with higher scores denoting worse HRQoL. Regarding its psychometric value, SLEQoL has been shown to have good internal consistency, test–retest reliability, construct validity and responsiveness. However, it is limited by its significant floor effects, whereby patients reported good perceived QoL beyond the instrument’s measurement capabilities. Authors suggested that this could be addressed through co-administering another validated PRO tool such as SF-36, although this would impose a higher time burden on both clinicians and patients [53]. To date, cross-cultural adaptation and validation of SLEQoL has been performed in Arabic (Egypt) [54], Thai (Thailand) [55], Chinese (China) [56] and Brazilian Portuguese (Brazil) [57].

### 2.4. L-QoL

L-QoL is another tool that serves to assess quality-of-life in SLE patients but on a needs-based approach [21,58]. It was originally developed and validated in the United Kingdom in 2008 and since then has only been translated and validated in a Turkish SLE cohort [59]. The tool comprises 25 questions which are answered in a “true/not true” response format. Summary scores range from 0 to 25, with higher scores indicating worse QoL. Content validity was achieved through items being generated via patient interviews and being predominantly phrased in their own words. In the original study, L-QoL also demonstrated excellent construct validity, internal consistency and test–retest reliability. However, construct validity was examined against non-validated self-reported measures of disease activity and severity. No validated physician assessments of these parameters were employed, and thus further studies including these will be required to further clarify the construct and discriminant validities of this tool [21,58]. Moreover, L-QoL has not been used in any clinical studies or research trials thus far, and its utility in patients with more severe disease phenotypes is to be explored [21].

### 2.5. LIT

The Lupus Impact Tracker (LIT) is a 10-item PRO tool that was designed with the aims of producing a simple but reliable PRO instrument to monitor the impact of SLE on the lives of patients over time [60,61]. The questions cover seven key concepts including cognition, lupus medications, physical health, pain/fatigue impact, emotional health, body image and planning desires and goals. The questions were generated using a multi-step approach that ultimately filtered out items with the highest psychometric value and strongest correlation with overall wellbeing/disease activity/damage scores and that ranked most importantly to patients from the 43-items of the LupusPRO. LIT was shown to have good internal consistency, responsiveness and test–retest reliability, but there are no data on floor and ceiling effects [14,60,61]. Cross-cultural validation has been displayed in Canada [62], south-eastern US [63] and five European countries including Germany, Italy, Spain, Sweden and France [64]. In addition, LIT was found to be a valid PRO tool in a multicultural Australian cohort and could distinguish between groups of patients with active or inactive disease [16]. Similar findings were also recently observed in a single-centre but ethnically diverse cohort of paediatric SLE patients [65]. In this paediatric study, LIT was deemed to be highly patient-feasible, given all patients (100%, *n* = 46) had completed their forms in all visits (115 in total) with accurate self-scoring. In correspondence, developers of the LIT reported that, among patients (pts) and physicians (phs) across 20 different centres, more than half agreed that LIT was not burdensome (>80% pts, >60% phs), helped foster better communication (>75% pts, >50% physicians) and facilitated discussion about the impact of SLE on QoL (>80% pts, >70% phs) [61].

## 3. GENERIC PRO Instruments

### 3.1. SF-36

The Medical Outcomes Study Short Form 36 (SF-36) is one of the most widely used generic HRQoL measurements in SLE. It consists of 36 question items grouped across 8 domains (physical functioning, general health, mental health, vitality, role physical, role emotional, bodily pain and social functioning) and can be expressed as two summary scores (physical and mental health component, PCS and MCS, respectively). Individual domain scores are then transformed into a scale ranging from 0 (worst) to 100 (best). As a generic questionnaire, it has the advantage of enabling comparisons with healthy population norms and other chronic diseases. This is particularly important, as patients with SLE have a significantly reduced QoL across all health domains when compared to other conditions [66].

The SF-36 has been extensively validated in various SLE populations over the last 25 years with good results [34,67,68,69,70]. As such, it has essentially become a gold standard among HRQoL instruments for validating other generic PRO tools such as PROMIS and MDHAQ. The SF-36 has been incorporated into clinical trials and randomised controlled trials (RCTs) to assess the efficacy of new therapies for SLE. For example, SF-36 scores were used as a major secondary end point in the BLISS trials, which facilitated the approval of belimumab after establishing its efficacy and safety in active SLE patients [71]. The SF-36 has remained the most widely used generic PRO instrument for clinical trials involving biologics, including rituximab [72,73], abatacept [74,75], cyclophosphamide [76], eprutuzumab [77] and sirukumab [78].

The need for a HRQoL tool as a key indicator in the routine clinical monitoring of SLE is becoming increasingly recognised in the literature [79,80]. Although not designed for clinical use, SF-36 remains an option, given its widespread use and international validation for a range of chronic diseases [79]. However, there are limited data regarding the actual use of SF-36 in routine clinical practice for SLE. A Canadian SLE clinic reported only minimal change in SF-36 scores over an 8-year period [81]. Despite the extensive validation and use in research settings, the use of the SF-36 in a purely clinical context remains limited.

Further studies examining the longitudinal responsiveness of SF-36 have yielded conflicting results [33,69,70,82]. One study demonstrated that social functioning and MCS scores were minimally responsive in patients with worsening disease damage [70]. Devilliers’ study in 2015 also showed that the LupusQoL was more responsive to changes in QoL than the SF-36 [33]. Given the complex multifaceted nature of SLE, it is not surprising that social and emotional nuances are perhaps more accurately captured and tracked by disease-specific or at least rheumatology-specific PRO tools. Furthermore, the SF-36 is relatively time-consuming to complete, and the scoring system is difficult in a busy clinical setting, requiring computer programming software. The SF-36 is likely to remain more appropriate in short- to medium-term clinical studies than in routine clinical use.

### 3.2. EQ-5D

The EuroQoL five-dimensional (EQ-5D) is a simple and standardised questionnaire which can yield clinical and economic data. It tackles the five domains of mobility, self-care, usual activities, pain and discomfort, and anxiety and depression using three-point response scales, which are then converted to a summary score from 0 (worst) to 1.0 (best). There is an additional visual analogue scale measuring the patient’s health perception from 0 (worst) to 100 (best). The EQ-5D has demonstrated favourable psychometric properties and exhibits satisfactory criterion validity, convergent validity and sensitivity to self-reported change in health [83]. Construct validity was also proven against equivalent domains in disease-specific PROs in a cohort of 240 patients; however, the same study also reported significant ceiling effects [84]. The EQ-5D has been used alongside the SF-36 in multiple clinical studies [85,86], as well as for comparison between rheumatic groups [87,88].

The EQ-5D has utility in forming economic appraisals rather than in just simply measuring HRQoL. Different utility values are generated from different health outcomes to calculate quality-adjusted life-years (QALYs) using the time trade-off method (TTO). This method has been validated against direct utility instruments in a cohort of 245 consecutive SLE patients in China [89]. As such, the EQ-5D has enabled economic appraisals and cost studies involving SLE patients [90,91,92]. For example, one Italian study demonstrated belimumab to be cost-effective (32,859 euros per QALY) [91]. However, it is unlikely that these benefits can be translated to a routine, patient-focused clinical setting.

### 3.3. PROMIS

PROMIS (Patient-Reported Outcomes Measurement Information System) is a relatively recent initiative developed by the NIH (National Institutes of Health) aimed at measuring PROs across various medical conditions [93]. It consists of question items from the eight core domains examining fatigue, pain intensity, pain interference, physical function, sleep disturbance, anxiety, depression and ability to participate in social roles and activities. Unlike conventional PRO tools, PROMIS also enables the application of the item response theory (IRT) and computerised adaptive testing (CAT) in order to develop calibrated item banks for more precise and efficient outcome measures [94].

PROMIS is not disease-specific, and as such, its content relevance to SLE needs to be examined. A study comprised of multi-ethnic English-speaking Asian individuals demonstrated that the eight core PROMIS domains largely aligned with the pertinent issues faced by patients with SLE [95]. However, this study also identified content gaps such as family burden, stigma and discrimination, although this may have been influenced by the demographics of the study cohort. There has been a paucity of further studies specifically analysing the content relevance of PROMIS to SLE in different populations.

One of the first studies to evaluate the validity of PROMIS in SLE was conducted in a childhood-onset SLE population. Most notably, it found that PROMIS demonstrated internal consistency and construct validity, despite taking less than five minutes to complete [96]. Since then, the body of literature has grown, with further studies in the adult SLE population showing similar results [97,98]. The California Lupus Epidemiology Study (CLUES) consisted of a racially and linguistically diverse cohort of 431 individuals. In this cohort, the PROMIS was able to demonstrate consistent reliability across racial/ethnic/language groups and was able to correlate well with the SF-36 [97]. Floor effects were minimal, and it was noted that ceiling effects were prevalent, especially in Social Health measures, which could adversely affect longitudinal effectiveness. The PROMIS has been shown to be sensitive to change in patient-reported improvement or worsening (effect size >|0.27|); however, this was only examined across physical and mental health domains [99]. Further studies are required to investigate the responsiveness to change in the social health domain.

One of the advantages of PROMIS is that it encompasses a wide variety of domains, despite placing a reduced burden on the patient. As such, PROMIS has been increasingly used in studies to investigate a range of SLE symptoms including pain [51], fatigue [100,101], depression [102], quality of life [103], cognitive impairment [104] and sleep quality [105]. Most notably, some of these symptoms such as cognitive impairment and sleep resonate strongly with SLE patients yet are known to be content gaps in other generic questionnaires [106]. Furthermore, the standardised metric of PROMIS also enables direct comparisons between SLE and other rheumatological or chronic conditions. Interestingly, the validity of PROMIS in fibromyalgia was found to be markedly lower when compared to OA, RA and SLE in a rheumatology cohort [107]. No studies to date have examined the use of PROMIS in concomitant fibromyalgia in SLE specifically. Further studies of PROMIS, including in RCTs and routine clinical care, are anticipated.

### 3.4. MDHAQ

The MDHAQ is a double-sided one-page questionnaire developed in rheumatology practice and contains six core measures. Pain, patient global and fatigue are scored on a 0–10 VAS, whereas function, joint count and symptom checklist are scored between 0 and 10, 0 and 48 and 0 and 60, respectively. Various scores can be calculated from the MDHAQ, including RAPID3, an index that incorporates three of the MDHAQ items, i.e., function, pain and patient global. The MDHAQ and RAPID3 are well validated in rheumatoid arthritis and other rheumatic diseases, including several studies supporting their utility in SLE. An American study supported the use of MDHAQ/RAPID3 in a cohort of 161 SLE patients in routine care [20]. The study reported robust internal consistency reliability (Cronbach’s alpha 0.88), validity and responsiveness to change for MDHAQ items and the RAPID3. However, the study noted significant floor effects compared to similar studies in the rheumatoid arthritis population. The RAPID3 will inherently align more strongly with the RA phenotype, where painful joints can significantly impact function, whereas the multisystem complexity of SLE may not translate as effectively to RAPID3 scores.

MDHAQ is rheumatology-specific rather than generic or SLE-specific, which gives it a unique advantage in examining the interplay between different rheumatic diseases. For example, it has been shown to be able to provide clues of concomitant fibromyalgia in SLE [18]. This is particularly important in the context of SLE, where there may be a high prevalence of non-inflammatory symptoms, characteristic of type 2 SLE. Recently, the concept of type 1 and type 2 SLE was proposed [108], whereby type 1 entails autoimmune and organ damage, whilst type 2 SLE is driven by symptoms typically observed in fibromyalgia. Type 2 symptoms of fatigue, myalgia, mood disturbance and cognitive function are typically not responsive to immunosuppression and thus crucial to recognise to avoid over-treatment and guide appropriate management.

The precise role of the MDHAQ in SLE is still yet to be fully established. The MDHAQ can be given to all patients in the waiting room of a rheumatology clinic regardless of their precise diagnosis, making it feasible in busy clinical settings. It is quick to complete and interpret in the clinic. It differs from typical HRQoL tools in that it has also been shown to reflect inflammatory disease activity and clinical improvement [19,109]. The utility of MDHAQ is multifaceted, much like SLE itself, as long as scores are interpreted in the context of traditional patient workups.

## 4. Discussion

There are numerous well-validated tools for measuring HRQoL in people with SLE, both disease-specific and generic (Table 1). These instruments have become essential in clinical trials of SLE, acknowledging that disease activity and damage are insufficient measures of the patient experience of living with the disease [13,14]. The use of an HRQoL tool has long been recommended by leading rheumatology groups including ACR [7], EULAR [8] and OMERACT [9] and indeed would seem mandatory for regulatory approval of new therapeutics. Despite their importance, there is no single instrument that is universally accepted as the gold standard for capturing every aspect of HRQoL in people with SLE. In general, the SF-36 and EQ-5D have been widely used due to their broad acceptance and application to numerous populations and diseases. Several SLE-specific tools have been used in the assessment of therapeutic strategies, with some evidence for increased sensitivity to change (when compared with SF36), making them appealing options in the clinical trial setting [13,14]. Further research is needed to determine the optimal instrument for assessing HRQoL in SLE.

Despite their widespread use in the research setting, measures of HRQoL remain underutilised in many clinical settings [11,15]. There are several real and perceived challenges to their use, including the complexity of the instruments, as well as the time and expertise needed to administer the instruments, calculate and interpret the results. These challenges have led to the development of more clinically focused tools, (such as MDHAQ [19,20]) and computerised adaptive questioning (PROMIS [93,94,95,96,103]) that sacrifice comprehensive assessment for the sake of practicality. Again, there is no single measure that is appropriate to all circumstances, though we would argue that despite this limitation, some form of measurement is better than none. Given that disease activity and damage may not capture the most prevalent and concerning symptoms that matter to people with SLE [3,4], it would seem prudent to attempt documentation of the patient’s concerns. More work is needed to determine which instruments best capture this and are sensitive to change whilst remaining practical and convenient for both the patient and the clinician.

The concept of ‘type 1’ and ‘type 2’ lupus symptoms has recently been proposed as a method of categorising symptoms and acknowledging the disparity between physician and patient assessment of SLE [17,108]. This proposal essentially acknowledges that ‘type 1’ symptoms (often considered inflammatory) are different from ‘type 2’ symptoms (fatigue, widespread pain, sleep disorders, depression and anxiety, frequently considered as fibromyalgia) that are prevalent in SLE. Incorporating PRO tools into patient management allows for documentation and validation of these symptoms, which may help bridge the gap between physician and patient assessment of the disease. Patient-reported instruments, interpreted by clinicians experienced in the care of people with SLE, aid in the recognition of fibromyalgia and type 2 symptoms, which may in turn allow for more accurate assessment of disease activity and inform treatment decisions. Whilst challenges remain in determining whether these measures accurately quantify severity and are sensitive to change, they seem sensible additions to patient-centred care.

In this review, we have highlighted the evolving role of PROs in the assessment of HRQoL in people with SLE, in both research and clinical settings. Many options now exist that have been validated in the trial setting, with increasing evidence for several PROMs in clinical practice. Further work is anticipated to better define the optimal tool for various clinical settings. Despite this limitation, integrating PROMs into clinical practice complements disease activity and damage measures and enhances patient-centred care.

## Figures and Tables

**Table 1:** Summary table of PRO instruments validated for use in SLE.

	HRQoL Tool	Country	Year	Purpose and Content	Time Burden	Number of Items	Response	Summary Score	Recall Period	Strengths	Weaknesses
**Generic**	**SF-36 [67]**	US	1990	Self-report measure QOL in various populations. Eight domains (physical functioning, general health, mental health, vitality, role physical, role emotional, bodily pain, and social functioning) and two summary scores (physical and mental health component)	Respondent: <10 min. Administration: <10 min.	36	3–6 point response scale	0–100 (higher scores = better QoL)	4 weeks	Most extensively studied and validated generic PRO tool in SLE. Has been used in multiple clinical trials. Facilitates comparison with other diseases.	Time-consuming and difficult scoring system. Questionable longitudinal responsiveness.
	**EQ-5D [83]**	UK	1994	Generic questionnaire for clinical and economic appraisal. Five dimensions of health: mobility, self-care, usual activities, pain and discomfort, anxiety and depression	Respondent: <5 min. Administration: <5 min	6	3-point response scale	0–100 (higher scores = better QoL)	1 day	Allows for economic evaluation. Has been used in clinical trials.	Encompasses fewer domains.
	**MDHAQ [20]**	US	1999	Assessment and monitoring of patients with rheumatic diseases	Respondent: <10 min. Administration: <5 min	4 core items	Variable—Visual analogue scores, checklists, 4-item response scales	0–10 for individual items (Function, Pain, Patient Global) Also utilises composite indices, e.g., RAPID3	Variable—Day (joint count) to week (function) to month (systems review)	Rheumatology-specific, allowing for analyses of specific sub-cohorts, e.g., concomitant fibromyalgia in SLE. A single double-sided page. Simple to calculate.	Minimally studied and validated in SLE cohorts.
	**PROMIS [95,103]**	US	2004	Standardised HRQoL measure. PROMIS29 covers eight domains (fatigue, pain intensity, pain interference, physical function, sleep disturbance, anxiety, depression and ability to participate in social roles and activities)	Respondent: <10 min. Administration: <10 min	Variable: 10 (PROMIS10), 29 (PROMIS29)	5-item response scale	T-score compared to US general population	1 week	Enables comparisons across a wide range of domains. Allows for item response theory and computer adaptive tests.	Significant ceiling effects. Difficult to score.
**SLE-Specific**	**LupusQOL [30]**	UK	2017	To measure disease-specific HRQoL in adult patients with SLE. It covers eight domains including physical health, emotional health, body image, pain, planning, fatigue, intimate relationships and burden to others	Respondent: <10 min. Administration: <5 min	34	5-point Likert response scale	0–100 (higher score = better QoL)	4 weeks	Most extensively studied and validated SLE-specific PRO Tool. Has been used in multiple clinical trials and studies. Has been translated and validated in the most languages of PRO tools.	Can be time-consuming.
	**LupusPRO [39]**	US	2012	To assess HRQoL in ethnically heterogeneous SLE populations and provide a gender-neutral PRO tool. Includes both HRQoL domains (lupus symptoms, lupus medications, physical health, emotional health, pain-vitality, procreation, cognition, body image) and non-HRQoL domains (desires/goals, social support, coping, satisfaction with care)	Respondent: Not reported. Administration: Not reported	43	5-point Likert response scale	0–100 (higher score = better QoL)	4 weeks	Was designed to accommodate ethnically heterogenous populations of SLE and also provides a gender-balanced PRO tool for SLE patients. Also encompasses non-HRQoL domains.	Has the most items of all SLE-Specific PRO tools and is thus time-consuming. Has not been widely used in clinical trials to date.
	**SLEQoL [53]**	Singapore	2005	To assess HRQoL in SLE patients. It covers six domains including physical functioning, activities, symptoms, treatment, mood and self-image.	Respondent: <5 min. Administration: Not reported	40	7-point response scale	40–280 (higher score = worst QoL)	1 week	Has good internal consistency, test–retest reliability, construct validity and responsiveness to change.	Significant floor effects, requires a second PRO tool to address this and is thus both time-consuming and less feasible. Has not been validated in more ethnically diverse SLE populations.
	**L-QoL [58]**	UK	2008	To assess HRQoL in SLE patients on a needs-based approach.	Respondent: <5 min. Administration: Not reported	25	Dichotomous. “True/Not True”	0–25 (higher score = worst QoL)	Nil (needs-based model)	Relatively simple and feasible to complete, given the dichotomous responses and lower item numbers. Constructed on a needs-based model.	Construct and discriminant validities need to be further explored. Has not been used in any clinical trials or SLE cohort studies to date.
	**LIT [60]**	US	2014	Short, simple and feasible PRO tool that monitors the impact of SLE over time	Respondent: <5 min. Administration:<5 min	10	5-point Likert response scale	0–100 (higher score = worst QoL)	4 weeks	Shortest, simplest and most feasible SLE-specific PRO tool that has been extensively validated. Demonstrates responsiveness to disease activity and can be used in paediatric SLE.	May miss certain aspects of HRQoL in SLE patients due to its brevity.

Abbreviations: EQ-5D; European Quality of Life Five-Dimensional, LIT; Lupus Impact Tracker, L-QoL; Lupus Quality of Life Questionnaire, LupusPRO; Lupus Patient-Reported Outcome, LupusQOL; Lupus Quality of Life, MDHAQ; Multi-Dimensional Health Assessment Questionnaire, PRO; Patient-Reported Outcomes, PROMIS; Patient-Reported Outcomes Measurement Information System, QOL; Quality of Life, SF-36; 36-item Short-Form Health Survey, SLE; Systemic Lupus Erythematosus, SLEQoL, Systemic Lupus Erythematosus Quality of Life.

## Abbreviations

ACR	American College of Rheumatology
EULAR	European Alliance of Associations for Rheumatology
EQ-5D	European Quality of Life Five-Dimensional
LIT	Lupus Impact Tracker
L-QoL	Lupus Quality of Life Questionnaire
LupusPRO	Lupus Patient-Reported Outcome
LupusQOL	Lupus Quality of Life
MDHAQ	Multi-Dimensional Health Assessment Questionnaire
OMERACT	Outcome Measures in Rheumatology Clinical Trials
PROMIS	Patient-Reported Outcomes Measurement Information System
SF-36	36-item Short-Form Health Survey
SLE	Systemic Lupus Erythematosus
SLEQoL	Systemic Lupus Erythematosus Quality of Life
WHO	World Health Organisation

## References

[1] Maidhof W., Hilas O. (2012). Lupus: An overview of the disease and management options. Pharm. Ther..

[2] McElhone K., Abbott J., Gray J., Williams A., Teh L.S. (2010). Patient perspective of systemic lupus erythematosus in relation to health-related quality of life concepts: A qualitative study. Lupus.

[3] Golder V., Ooi J.J., Antony A.S., Ko T., Morton S., Kandane-Rathnayake R., Morand E.F., Hoi A.Y. (2018). Discordance of patient and physician health status concerns in systemic lupus erythematosus. Lupus.

[4] Elera-Fitzcarrald C., Vega K., Gamboa-Cárdenas R.V., Zúñiga K., Medina M., Pimentel-Quiroz V., Pastor-Asurza C., Perich-Campos R., Bellido Z.R., Griffin R. (2020). Discrepant perception of lupus disease activity: A comparison between patients’ and physicians’ disease activity scores. J. Clin. Rheumatol..

[5] Julian L.J., Yelin E., Yazdany J., Panopalis P., Trupin L., Criswell L.A., Katz P. (2009). Depression, medication adherence, and service utilization in systemic lupus erythematosus. Arthritis Care Res..

[6] Bennett J.K., Fuertes J.N., Keitel M., Phillips R. (2011). The role of patient attachment and working alliance on patient adherence, satisfaction, and health-related quality of life in lupus treatment. Patient Educ. Couns..

[7] Ad Hoc Committee on Systemic Lupus Erythematosus Response Criteria for Fatigue (2007). Measurement of fatigue in systemic lupus erythematosus: A systematic review. Arthritis Care Res..

[8] Castrejon I., Carmona L., Agrinier N., Andres M., Briot K., Caron M., Christensen R., Consolaro A., Curbelo R., Ferrer M. (2015). The EULAR Outcome Measures Library: Development and an example from a systematic review for systemic lupus erythematous instruments. Clin. Exp. Rheumatol..

[9] Strand V., Gladman D., Isenberg D., Petri M., Smolen J., Tugwell P. (2000). Endpoints: Consensus recommendations from OMERACT IV. Lupus.

[10] Kwan A., Strand V., Touma Z. (2017). The role of patient-reported outcomes in systemic lupus erythematosus. Curr. Treat. Options Rheumatol..

[11] Dua A.B., Touma Z., Toloza S., Jolly M. (2013). Top 10 recent developments in health-related quality of life in patients with systemic lupus erythematosus. Curr. Rheumatol. Rep..

[12] Thumboo J., Strand V. (2007). Health-related quality of life in patients with systemic lupus erythematosus: An update. Ann. Acad. Med. Singap..

[13] Mahieu M., Yount S., Ramsey-Goldman R. (2016). Patient-reported outcomes in systemic lupus erythematosus. Rheum. Dis. Clin..

[14] Izadi Z., Gandrup J., Katz P.P., Yazdany J. (2018). Patient-reported outcome measures for use in clinical trials of SLE: A review. Lupus Sci. Med..

[15] Castrejón I., Tani C., Jolly M., Huang A., Mosca M. (2014). Indices to assess patients with systemic lupus erythematosus in clinical trials, long-term observational studies, and clinical care. Clin. Exp. Rheumatol..

[16] Antony A., Kandane-Rathnayake R.K., Ko T., Boulos D., Hoi A.Y., Jolly M., Morand E.F. (2017). Validation of the Lupus Impact Tracker in an Australian patient cohort. Lupus.

[17] Rogers J.L., Eudy A.M., Pisetsky D., Criscione-Schreiber L.G., Sun K., Doss J., Clowse M.E. (2021). Using Clinical Characteristics and Patient-Reported Outcome Measures to Categorize Systemic Lupus Erythematosus Subtypes. Arthritis Care Res..

[18] Huang F.F., Fang R., Nguyen M.H., Bryant K., Gibson K.A., O’Neill S.G. (2020). Identifying co-morbid fibromyalgia in patients with systemic lupus erythematosus using the Multi-Dimensional Health Assessment Questionnaire. Lupus.

[19] Askanase A.D., Castrejón I., Pincus T. (2011). Quantitative data for care of patients with systemic lupus erythematosus in usual clinical settings: A patient Multidimensional Health Assessment Questionnaire and physician estimate of noninflammatory symptoms. J. Rheumatol..

[20] Annapureddy N., Giangreco D., Devilliers H., Block J.A., Jolly M. (2018). Psychometric properties of MDHAQ/RAPID3 in patients with systemic lupus erythematosus. Lupus.

[21] Yazdany J. (2011). Health-related quality of life measurement in systemic lupus erythematosus: The LupusQoL, SLEQoL, and L-QoL. Arthritis Care Res..

[22] Jolly M., Pickard A.S., Wilke C., Mikolaitis R.A., Teh L.S., Mcelhone K., Fogg L., Block J. (2010). Lupus-specific health outcome measure for US patients: The LupusQoL-US version. Ann. Rheum. Dis..

[23] González-Rodríguez V., Peralta-Ramírez M.I., Navarrete-Navarrete N., Callejas-Rubio J.L., Ruiz S.A.M., Khamashta M. (2009). Adaptation and validation of the Spanish version of a disease-specific quality of life measure in patients with systemic lupus erythematosus: The Lupus quality of life. Med. Clin..

[24] Hosseini N., Bonakdar Z.S., Gholamrezaei A., Mirbagher L. (2014). Linguistic validation of the LupusQoL for the assessment of quality of life in Iranian patients with systemic lupus erythematosus. Int. J. Rheumatol..

[25] Pamuk O.N., Onat A.M., Donmez S., Mengüs C., Kisacik B. (2015). Validity and reliability of the Lupus QoL index in Turkish systemic lupus erythematosus patients. Lupus.

[26] Conti F., Perricone C., Reboldi G., Gawlicki M., Bartosiewicz I., Pacucci V.A., Massaro L., Miranda F., Truglia S., Alessandri C. (2014). Validation of a disease-specific health-related quality of life measure in adult Italian patients with systemic lupus erythematosus: LupusQoL-IT. Lupus.

[27] Devilliers H., Amoura Z., Besancenot J.F., Bonnotte B., Pasquali J.L., Wahl D., Maurier F., Kaminsky P., Pennaforte J.L., Magy-Bertrand N. (2012). LupusQoL-FR is valid to assess quality of life in patients with systemic lupus erythematosus. Rheumatology.

[28] Carrión-Nessi F.S., Marcano-Rojas M.V., Freitas-DeNobrega D.C., Arocha S.R.R., Antuarez-Magallanes A.W., Fuentes-Silva Y.J. (2021). Validation of the LupusQoL in Venezuela: A Specific Measurement of Quality of Life in Patients with Systemic Lupus Erythematosus. Reumatol. Clin..

[29] Wang S.L., Wu B., Leng L., Bucala R., Lu L.J. (2013). Validity of LupusQoL-China for the assessment of health related quality of life in Chinese patients with systemic lupus erythematosus. PLoS ONE.

[30] McElhone K., Abbott J., Shelmerdine J., Bruce I.N., Ahmad Y., Gordon C., Peers K., Isenberg D., Ferenkeh-Koroma A., Griffiths B. (2007). Development and validation of a disease-specific health-related quality of life measure, the LupusQoL, for adults with systemic lupus erythematosus. Arthritis Care Res..

[31] Louthrenoo W., Kasitanon N., Morand E., Kandane-Rathnayake R. (2020). Comparison of performance of specific (SLEQOL) and generic (SF36) health-related quality of life questionnaires and their associations with disease status of systemic lupus erythematosus: A longitudinal study. Arthritis Res. Ther..

[32] McElhone K., Abbott J., Sutton C., Mullen M., Lanyon P., Rahman A., Yee C.S., Akil M., Bruce I.N., Ahmad Y. (2016). Sensitivity to change and minimal important differences of the LupusQoL in patients with systemic lupus erythematosus. Arthritis Care Res..

[33] Devilliers H., Amoura Z., Besancenot J.F., Bonnotte B., Pasquali J.L., Wahl D., Maurier F., Kaminsky P., Pennaforte J.L., Magy-Bertrand N. (2015). Responsiveness of the 36-item Short Form Health Survey and the Lupus Quality of Life questionnaire in SLE. Rheumatology.

[34] Nantes S.G., Strand V., Su J., Touma Z. (2018). Comparison of the Sensitivity to Change of the 36-Item Short Form Health Survey and the Lupus Quality of Life Measure Using Various Definitions of Minimum Clinically Important Differences in Patients with Active Systemic Lupus Erythematosus. Arthritis Care Res..

[35] Clowse M.E., Wallace D.J., Furie R.A., Petri M.A., Pike M.C., Leszczyński P., Neuwelt C.M., Hobbs K., Keiserman M., Duca L. (2017). Efficacy and safety of epratuzumab in moderately to severely active systemic lupus erythematosus: Results from two phase III randomized, double-blind, placebo-controlled trials. Arthritis Rheumatol..

[36] Askanase A.D., Wan G.J., Panaccio M.P., Zhao E., Zhu J., Bilyk R., Furie R.A. (2021). Patient-Reported Outcomes from a Phase 4, Multicenter, Randomized, Double-Blind, Placebo-Controlled Trial of Repository Corticotropin Injection (Acthar^®^ Gel) for Persistently Active Systemic Lupus Erythematosus. Rheumatol. Ther..

[37] Keramiotou K., Anagnostou C., Kataxaki E., Galanos A., Sfikakis P.P., Tektonidou M.G. (2020). The impact of upper limb exercise on function, daily activities and quality of life in systemic lupus erythematosus: A pilot randomised controlled trial. RMD Open..

[38] Khan F., Granville N., Malkani R., Chathampally Y. (2020). Health-Related Quality of Life Improvements in Systemic Lupus Erythematosus Derived from a Digital Therapeutic Plus Tele-Health Coaching Intervention: Randomized Controlled Pilot Trial. J. Med. Internet Res..

[39] Jolly M., Pickard A.S., Block J.A., Kumar R.B., Mikolaitis R.A., Wilke C.T., Rodby R.A., Fogg L., Sequeira W., Utset T.O. (2012). Disease-specific patient reported outcome tools for systemic lupus erythematosus. Semin. Arthritis Rheum..

[40] Navarra S., Tanangunan R.M., Mikolaitis-Preuss R.A., Kosinski M., Block J.A., Jolly M. (2013). Cross-cultural validation of a disease-specific patient-reported outcome measure for lupus in Philippines. Lupus.

[41] Kaya A., Goker B., Cura E.S., Tezcan M.E., Tufan A., Mercan R., Bitik B., Haznedaroglu S., Ozturk M.A., Mikolaitis-Preuss R.A. (2014). Turkish lupusPRO: Cross-cultural validation study for Lupus. Clin. Rheumatol..

[42] Jolly M., Toloza S., Block J., Mikolaitis R., Kosinski M., Wallace D., Durran-Barragan S., Bertoli A., Blazevic I., Vilá L. (2013). Spanish LupusPRO: Cross-cultural validation study for lupus. Lupus.

[43] Bourré-Tessier J., Clarke A.E., Kosinski M., Mikolaitis-Preuss R.A., Bernatsky S., Block J.A., Jolly M. (2014). The French-Canadian validation of a disease-specific, patient-reported outcome measure for lupus. Lupus.

[44] Jolly M., Mazzoni D., Cicognani E. (2015). Measurement properties of Web based Lupuspro, a disease targeted Outcome Tool, among Italian Patients with Lupus. Value Health.

[45] Inoue M., Shiozawa K., Yoshihara R., Yamane T., Shima Y., Hirano T., Jolly M., Makimoto K. (2017). The Japanese LupusPRO: A cross-cultural validation of an outcome measure for lupus. Lupus.

[46] Pinto B., Jolly M., Dhooria A., Grover S., Raj J.M., Devilliers H., Sharma A. (2019). Hindi LupusPRO: Cross cultural validation of disease specific patient reported outcome measure of lupus. Lupus.

[47] Elkaraly N.E., Nasef S.I., Omar A.S., Fouad A.M., Jolly M., Mohamed A.E. (2020). The Arabic LupusPRO: A Cross-Cultural Validation of a Disease-Specific Patient-Reported Outcome Tool for Quality of Life in Lupus Patients. Lupus.

[48] Mok C.C., Kosinski M., Ho L.Y., Chan K.L., Jolly M. (2015). Validation of the LupusPRO in Chinese patients from Hong Kong with systemic lupus erythematosus. Arthritis Care Res..

[49] Azizoddin D.R., Olmstead R., Cost C., Jolly M., Ayeroff J., Racaza G., Sumner L.A., Ormseth S., Weisman M., Nicassio P.M. (2017). A multi-group confirmatory factor analyses of the LupusPRO between southern California and Filipino samples of patients with systemic lupus erythematosus. Lupus.

[50] Azizoddin D.R., Weinberg S., Gandhi N., Arora S., Block J.A., Sequeira W., Jolly M. (2018). Validation of the LupusPRO version 1.8: An update to a disease-specific patient-reported outcome tool for systemic lupus erythematosus. Lupus.

[51] Allen K.D., Beauchamp T., Rini C., Keefe F.J., Bennell K.L., Cleveland R.J., Grimm K., Huffman K., Hu D.G., Santana A. (2021). Pilot study of an internet-based pain coping skills training program for patients with systemic Lupus Erythematosus. BMC Rheumatol..

[52] Jolly M., Peters K.F., Mikolaitis R., Evans-Raoul K., Block J.A. (2014). Body image intervention to improve health outcomes in lupus: A pilot study. JCR J. Clin. Rheumatol..

[53] Leong K.P., Kong K.O., Thong B.Y., Koh E.T., Lian T.Y., Teh C.L., Cheng Y.K., Chng H.H., Badsha H., Law W.G. (2005). Development and preliminary validation of a systemic lupus erythematosus-specific quality-of-life instrument (SLEQOL). Rheumatology.

[54] Aziz M.M., Galal M.A., Elzohri M.H., El-Nouby F., Leong K. (2018). Cross-cultural adaptation and validation of Systemic Lupus Erythematosus Quality of Life questionnaire into Arabic. Lupus.

[55] Kasitanon N., Wangkaew S., Puntana S., Sukitawut W., Leong K.P., Louthrenoo W., Tan Tock Seng Hospital Lupus Study Group (2013). The reliability, validity and responsiveness of the Thai version of Systemic Lupus Erythematosus Quality of Life (SLEQOL-TH) instrument. Lupus.

[56] Jiang H.Z., Lin Z.G., Li H.J., Wang S.Y., Guan S.Q., Mei Y.F. (2018). The Chinese version of the SLEQOL is a reliable assessment of health-related quality of life in Han Chinese patients with systemic lupus erythematosus. Clin. Rheumatol..

[57] Freire E.A., Bruscato A., Leite D.R., Sousa T.T., Ciconelli R.M. (2010). Translation into Brazilian Portuguese, cultural adaptation and validatation of the systemic lupus erythematosus quality of life questionnaire (SLEQOL). Acta Reumatol. Port..

[58] Doward L.C., McKenna S.P., Whalley D., Tennant A., Griffiths B., Emery P., Veale D.J. (2009). The development of the L-QoL: A quality-of-life instrument specific to systemic lupus erythematosus. Ann. Rheum. Dis..

[59] Duruöz M.T., Unal C., Toprak C.S., Sezer I., Yilmaz F., Ulutatar F., Atagündüz P., Baklacioglu H.S. (2017). The validity and reliability of Systemic Lupus Erythematosus Quality of Life Questionnaire (L-QoL) in a Turkish population. Lupus.

[60] Jolly M., Garris C.P., Mikolaitis R.A., Jhingran P.M., Dennis G., Wallace D.J., Clarke A., Dooley M.A., Parke A., Strand V. (2014). Development and validation of the Lupus Impact Tracker: A patient-completed tool for clinical practice to assess and monitor the impact of systemic lupus erythematosus. Arthritis Care Res..

[61] Jolly M., Kosinski M., Garris C.P., Oglesby A.K. (2016). Prospective validation of the Lupus Impact Tracker: A patient-completed tool for clinical practice to evaluate the impact of systemic lupus erythematosus. Arthritis Rheumatol..

[62] Bourré-Tessier J., Clarke A.E., Mikolaitis-Preuss R.A., Kosinski M., Bernatsky S., Block J.A., Jolly M. (2013). Cross-cultural validation of a disease-specific patient-reported outcome measure for systemic lupus erythematosus in Canada. J. Rheumatol..

[63] Brandt J.E., Drenkard C., Kan H., Bao G., Dunlop-Thomas C., Pobiner B., Chang D.J., Jolly M., Lim S.S. (2017). External validation of the lupus impact tracker in a southeastern US longitudinal cohort with systemic lupus erythematosus. Arthritis Care Res..

[64] Schneider M., Mosca M., Pego-Reigosa J.M., Gunnarsson I., Maurel F., Garofano A., Perna A., Porcasi R., Devilliers H. (2017). Cross-cultural validation of Lupus Impact Tracker in five European clinical practice settings. Rheumatology.

[65] Ganguli S.K., Hui-Yuen J.S., Jolly M., Cerise J., Eberhard B.A. (2020). Performance and psychometric properties of lupus impact tracker in assessing patient-reported outcomes in pediatric lupus: Report from a pilot study. Lupus.

[66] Jolly M. (2005). How does quality of life of patients with systemic lupus erythematosus compare with that of other common chronic illnesses?. J. Rheumatol..

[67] Stoll T., Gordon C., Seifert B., Richardson K., Malik J., Bacon P.A., Isenberg D.A. (1997). Consistency and validity of patient administered assessment of quality of life by the MOS SF-36; its association with disease activity and damage in patients with systemic lupus erythematosus. J. Rheumatol..

[68] Thumboo J., Fong K.Y., Ng T.P., Leong K.H., Feng P.H., Thio S.T., Boey M.L. (1999). Validation of the MOS SF-36 for quality of life assessment of patients with systemic lupus erythematosus in Singapore. J. Rheumatol..

[69] Touma Z., Gladman D.D., Ibañez D., Urowitz M.B. (2011). Is there an advantage over SF-36 with a quality of life measure that is specific to systemic lupus erythematosus?. J. Rheumatol..

[70] Baba S., Katsumata Y., Okamoto Y., Kawaguchi Y., Hanaoka M., Kawasumi H., Yamanaka H. (2018). Reliability of the SF-36 in Japanese patients with systemic lupus erythematosus and its associations with disease activity and damage: A two-consecutive year prospective study. Lupus.

[71] Furie R., Petri M., Zamani O., Cervera R., Wallace D.J., Tegzová D., Sanchez-Guerrero J., Schwarting A., Merrill J.T., Chatham W.W. (2011). A phase III, randomized, placebo-controlled study of belimumab, a monoclonal antibody that inhibits B lymphocyte stimulator, in patients with systemic lupus erythematosus. Arthritis Rheum..

[72] Merrill J.T., Neuwelt C.M., Wallace D.J., Shanahan J.C., Latinis K.M., Oates J.C., Utset T.O., Gordon C., Isenberg D.A., Hsieh H.J. (2010). Efficacy and safety of rituximab in moderately-to-severely active systemic lupus erythematosus: The randomized, double-blind, phase II/III systemic lupus erythematosus evaluation of rituximab trial. Arthritis Rheum..

[73] Rovin B.H., Furie R., Latinis K., Looney R.J., Fervenza F.C., Sanchez-Guerrero J., Maciuca R., Zhang D., Garg J.P., Brunetta P. (2012). Efficacy and safety of rituximab in patients with active proliferative lupus nephritis: The Lupus Nephritis Assessment with Rituximab study. Arthritis Rheum..

[74] Merrill J.T., Burgos-Vargas R., Westhovens R., Chalmers A., D’cruz D., Wallace D.J., Bae S.C., Sigal L., Becker J.C., Kelly S. (2010). The efficacy and safety of abatacept in patients with non–life-threatening manifestations of systemic lupus erythematosus: Results of a twelve-month, multicenter, exploratory, phase IIb, randomized, double-blind, placebo-controlled trial. Arthritis Rheum..

[75] Furie R., Nicholls K., Cheng T.T., Houssiau F., Burgos-Vargas R., Chen S.L., Hillson J.L., Meadows-Shropshire S., Kinaszczuk M., Merrill J.T. (2014). Efficacy and safety of abatacept in lupus nephritis: A twelve-month, randomized, double-blind study. Arthritis Rheumatol..

[76] ACCESS Trial Group (2014). Treatment of lupus nephritis with abatacept: The abatacept and cyclophosphamide combination efficacy and safety study. Arthritis Rheumatol..

[77] Wallace D.J., Kalunian K., Petri M.A., Strand V., Houssiau F.A., Pike M., Kilgallen B., Bongardt S., Barry A., Kelley L. (2014). Efficacy and safety of epratuzumab in patients with moderate/severe active systemic lupus erythematosus: Results from EMBLEM, a phase IIb, randomised, double-blind, placebo-controlled, multicentre study. Ann. Rheum. Dis..

[78] Thorne C., Takeuchi T., Karpouzas G.A., Sheng S., Kurrasch R., Fei K., Hsu B. (2018). Investigating sirukumab for rheumatoid arthritis: 2-year results from the phase III SIRROUND-D study. RMD Open.

[79] Fernando M.M., Isenberg D.A. (2005). How to monitor SLE in routine clinical practice. Ann. Rheum. Dis..

[80] Mosca M., Tani C., Aringer M., Bombardieri S., Boumpas D., Cervera R., Doria A., Jayne D., Khamashta M.A., Kuhn A. (2011). Development of quality indicators to evaluate the monitoring of SLE patients in routine clinical practice. Autoimmun. Rev..

[81] Kuriya B., Gladman D.D., Ibañez D., Urowitz M.B. (2008). Quality of life over time in patients with systemic lupus erythematosus. Arthritis Care Res..

[82] Hanly J.G., Urowitz M.B., Jackson D., Bae S.C., Gordon C., Wallace D.J., Clarke A., Bernatsky S., Vasudevan A., Isenberg D. (2011). SF-36 summary and subscale scores are reliable outcomes of neuropsychiatric events in systemic lupus erythematosus. Ann. Rheum. Dis..

[83] Aggarwal R., Wilke C.T., Pickard A.S., Vats V., Mikolaitis R., Fogg L., Block J.A., Jolly M. (2009). Psychometric properties of the EuroQol-5D and Short Form-6D in patients with systemic lupus erythematosus. J. Rheumatol..

[84] Wang S.L., Wu B., Zhu L.A., Leng L., Bucala R., Lu L.J. (2014). Construct and criterion validity of the Euro Qol-5D in patients with systemic lupus erythematosus. PLoS ONE.

[85] Parodis I., Lopez Benavides A.H., Zickert A., Pettersson S., Möller S., Welin Henriksson E., Voss A., Gunnarsson I. (2019). The Impact of Belimumab and Rituximab on Health-Related Quality of Life in Patients with Systemic Lupus Erythematosus. Arthritis Care Res..

[86] Shi Y., Li M., Liu L., Wang Z., Wang Y., Zhao J., Wang Q., Tian X., Li M., Zeng X. (2020). Relationship between disease activity, organ damage and health-related quality of life in patients with systemic lupus erythematosus: A systemic review and meta-analysis. Autoimmun. Rev..

[87] Park E.H., Strand V., Oh Y.J., Song Y.W., Lee E.B. (2019). Health-related quality of life in systemic sclerosis compared with other rheumatic diseases: A cross-sectional study. Arthritis Res. Ther..

[88] Chen H.H., Chen D.Y., Chen Y.M., Lai K.L. (2017). Health-related quality of life and utility: Comparison of ankylosing spondylitis, rheumatoid arthritis, and systemic lupus erythematosus patients in Taiwan. Clin. Rheumatol..

[89] Wang S.L., Hsieh E., Zhu L.A., Wu B., Lu L.J. (2015). Comparative assessment of different health utility measures in systemic lupus erythematosus. Sci. Rep..

[90] Ivorra J.R., Fernández-Llanio-Comella N., San-Martín-Álvarez A., Vela-Casasempere P., Saurí-Ferrer I., González-de-Julián S., Vivas-Consuelo D. (2019). Health-related quality of life in patients with systemic lupus erythematosus: A Spanish study based on patient reports. Clin. Rheumatol..

[91] Pierotti F., Palla I., Treur M., Pippo L., Turchetti G. (2015). Assessment of the economic impact of belimumab for the treatment of systemic lupus erythematosus in the Italian setting: A cost-effectiveness analysis. PLoS ONE.

[92] Bexelius C., Wachtmeister K., Skare P., Jönsson L., Vollenhoven R.V. (2013). Drivers of cost and health-related quality of life in patients with systemic lupus erythematosus (SLE): A Swedish nationwide study based on patient reports. Lupus.

[93] Cella D., Yount S., Rothrock N., Gershon R., Cook K., Reeve B., Ader D., Fries J.F., Bruce B., Rose M. (2007). The Patient-Reported Outcomes Measurement Information System (PROMIS): Progress of an NIH Roadmap cooperative group during its first two years. Med. Care.

[94] Fries J.F., Witter J., Rose M., Cella D., Khanna D., Morgan-DeWitt E. (2014). Item response theory, computerized adaptive testing, and PROMIS: Assessment of physical function. J. Rheumatol..

[95] Ow Y.L., Thumboo J., Cella D., Cheung Y.B., Yong Fong K., Wee H.L. (2011). Domains of health-related quality of life important and relevant to multiethnic English-speaking Asian systemic lupus erythematosus patients: A focus group study. Arthritis Care Res..

[96] Jones J.T., Carle A.C., Wootton J., Liberio B., Lee J., Schanberg L.E., Ying J., Morgan DeWitt E., Brunner H.I. (2017). Validation of Patient-Reported Outcomes Measurement Information System Short Forms for use in childhood-onset systemic lupus erythematosus. Arthritis Care Res..

[97] Katz P., Yazdany J., Trupin L., Rush S., Helmick C.G., Murphy L.B., Lanata C., Criswell L.A., Dall’Era M. (2019). Psychometric evaluation of the National Institutes of Health Patient-Reported Outcomes Measurement Information System in a multiracial, multiethnic systemic lupus erythematosus cohort. Arthritis Care Res..

[98] Kasturi S., Burket J.C., Berman J.R., Kirou K.A., Levine A.B., Sammaritano L.R., Mandl L.A. (2018). Feasibility of Patient-Reported Outcomes Measurement Information System (PROMIS^®^) computerized adaptive tests in systemic lupus erythematosus outpatients. Lupus.

[99] Kasturi S., Szymonifka J., Berman J.R., Kirou K.A., Levine A.B., Sammaritano L.R., Mandl L.A. (2020). Responsiveness of the Patient-Reported Outcomes Measurement Information System Global Health Short Form in Outpatients with Systemic Lupus Erythematosus. Arthritis Care Res..

[100] Mahieu M.A., Ahn G.E., Chmiel J.S., Dunlop D.D., Helenowski I.B., Semanik P., Song J., Yount S., Chang R.W., Ramsey-Goldman R. (2018). Serum adipokine levels and associations with patient-reported fatigue in systemic lupus erythematosus. Rheumatol. Int..

[101] Mahieu M.A., Ahn G.E., Chmiel J.S., Dunlop D.D., Helenowski I.B., Semanik P., Song J., Yount S., Chang R.W., Ramsey-Goldman R. (2016). Fatigue, patient reported outcomes, and objective measurement of physical activity in systemic lupus erythematosus. Lupus.

[102] Dietz B., Katz P., Dall’Era M., Murphy L.B., Lanata C., Trupin L., Criswell L.A., Yazdany J. (2021). Major Depression and Adverse Patient-Reported Outcomes in Systemic Lupus Erythematosus: Results from a Prospective Longitudinal Cohort. Arthritis Care Res..

[103] Lai J.S., Beaumont J.L., Jensen S.E., Kaiser K., Van Brunt D.L., Kao A.H., Chen S.Y. (2017). An evaluation of health-related quality of life in patients with systemic lupus erythematosus using PROMIS and Neuro-QoL. Clin. Rheumatol..

[104] Kim M.Y., Sen D., Drummond R.R., Brandenburg M.C., Biesanz K.L., Kim A.H., Eisen S.A., Baum C.M., Foster E.R. (2021). Cognitive dysfunction among people with systemic lupus erythematosus is associated with reduced participation in daily life. Lupus.

[105] Charoenwoodhipong P., Harlow S.D., Marder W., Hassett A.L., McCune W.J., Gordon C., Helmick C.G., Barbour K.E., Wang L., Mancuso P. (2020). Dietary Omega Polyunsaturated Fatty Acid Intake and Patient-Reported Outcomes in Systemic Lupus Erythematosus: The Michigan Lupus Epidemiology and Surveillance Program. Arthritis Care Res..

[106] Stamm T.A., Bauernfeind B., Coenen M., Feierl E., Mathis M., Stucki G., Smolen J.S., Machold K.P., Aringer M. (2007). Concepts important to persons with systemic lupus erythematosus and their coverage by standard measures of disease activity and health status. Arthritis Care Res..

[107] Katz P., Pedro S., Michaud K. (2017). Performance of the patient-reported outcomes measurement information system 29-item profile in rheumatoid arthritis, osteoarthritis, fibromyalgia, and systemic lupus erythematosus. Arthritis Care Res..

[108] Pisetsky D.S., Clowse M.E., Criscione-Schreiber L.G., Rogers J.L. (2019). A novel system to categorize the symptoms of systemic lupus erythematosus. Arthritis Care Res..

[109] Castrejón I., Bergman M.J., Pincus T. (2013). MDHAQ/RAPID3 to recognize improvement over 2 months in usual care of patients with osteoarthritis, systemic lupus erythematosus, spondyloarthropathy, and gout, as well as rheumatoid arthritis. J. Clin. Rheumatol..

